# Broadband spin-multiplexed single-celled metasurface holograms: a comprehensive comparison between different strategies

**DOI:** 10.1515/nanoph-2022-0535

**Published:** 2023-01-09

**Authors:** Sören im Sande, Sergey I. Bozhevolnyi, Fei Ding

**Affiliations:** Centre for Nano Optics, University of Southern Denmark, Campusvej 55, Odense M DK-5230, Denmark

**Keywords:** broadband, gap-surface plasmon, metasurface holography, single-celled geometric metasurfaces, spin-multiplexed

## Abstract

Metasurface-generated holograms have emerged as a unique platform for arbitrarily shaping the reflected/transmitted wavefronts with the advantages of subwavelength large pixel sizes and multiple information channels. However, achieving multiple holographic images with large operation bandwidths is a rather complicated and arduous issue due to the dissimilar dispersion of all meta-atoms involved. In this work, we design and experimentally demonstrate single-celled metasurfaces to realize broadband and spin-multiplexed holograms, whose phase modulation is based only on the geometric phase supplied by a judiciously designed high-performance nanoscale half-wave plate operating in reflection. Four different multiplexing strategies are implemented, and the resulting holograms are systemically assessed and compared with respect to background levels, image fidelities, holograms efficiencies, and polarization conversion ratios. Our work complements the methodologies available for designing multiplexed meta-holograms with versatile functionalities.

## 1 Introduction

Holography has numerous applications in sensing [1, 2], information encryption [3], [4], [5], information storage [5], and display technologies due to its versatile capabilities of image formation via arbitrary wavefront shaping [3]. The development of computer-generated holograms (CGHs), which are based on digitally computed and subsequently printed holographic interference patterns, has significantly enriched holography by dispensing with the need for complex setups to record the phase and amplitude of real objects [6]. However, the usage of conventional CGHs requires bulky components (e.g., spatial light modulators) to realize the required phase modulation, suffering thereby from limited channels and large pixel sizes [7].

Recently, optical metasurfaces have shown the unprecedented capability to manipulate the phase, amplitude, and polarization of light on the subwavelength scale with multiple degrees of freedom [8], [9], [10], [11], making them ideally suitable for encoding holograms, especially phase-only holograms [12], [13], [14], [15], [16], [17]. The available functionalities of phase-only meta-holograms are largely determined by the phase modulation methods used for designing meta-atoms that can be classified into the resonance and geometric phases [7, 8]. The resonance phase modulation mainly relies on adjusting the shapes and dimensions of meta-atoms in the vicinity of their resonances. To identify many meta-atoms featuring sufficiently large phase variations while maintaining equally high amplitudes, a tedious design procedure should be conducted. Although machine learning and other optimization algorithms could be employed to design free-form meta-atoms securing larger phase variations as compared to the intuitive designs (e.g., bricks [9], discs [18], and crosses [9, 19]), the resulting meta-atoms exhibit rather complex shapes, becoming really challenging to fabricate. Additionally, there is always a trade-off between the bandwidth and phase discretization due to the dissimilar dispersion of all meta-atoms involved [20]. Furthermore, the performance of differently sized or shaped meta-atoms located close to each other would suffer from non-negligible near-field coupling, strongly distorting the phase distributions expected from the performance of periodic meta-atom arrays considered in the design procedure [21]. In contrast to the resonance phase modulation, the geometric or Pancharatnam–Berry phase modulation is achieved with orientation-varied single-celled meta-atoms, enabling thereby complete and continuous phase control over the circularly polarized (CP) light without any dispersion, proving to be an efficient method to realize meta-holograms [3, 22, 23]. However, the geometric phase is intrinsically spin-conjugated, a circumstance that limits the number of information channels [24] as the opposite spin lacks the phase modulation. Even though the spin-decoupled metasurfaces have been proposed to circumvent this issue by combining both resonance and geometric phases, multiple meta-atoms that function as half-wave plates (HWPs) are required, which in turn comes at the expense of complex meta-atom design, constrained bandwidths, and low efficiencies. Therefore, it is highly desired to develop *broadband* meta-holograms featuring at the same time multiple information channels for incident CP waves with different spins.

In this work, we propose single-celled metasurfaces to realize broadband and spin-multiplexed holograms, in which the phase modulation relies only on the geometric phase supplied by a carefully designed high-performance nano-HWP operating in reflection, and conduct a systemic and comprehensive comparison between four different multiplexing approaches. By analyzing the background levels, image fidelities, holograms efficiencies, and polarization conversion ratios (PCRs) of all experimentally obtained holographic images, we develop a simple but effective design methodology for spin-decoupled meta-holograms with single-sized meta-atoms.

## 2 Results and discussion

**Figure 1:**
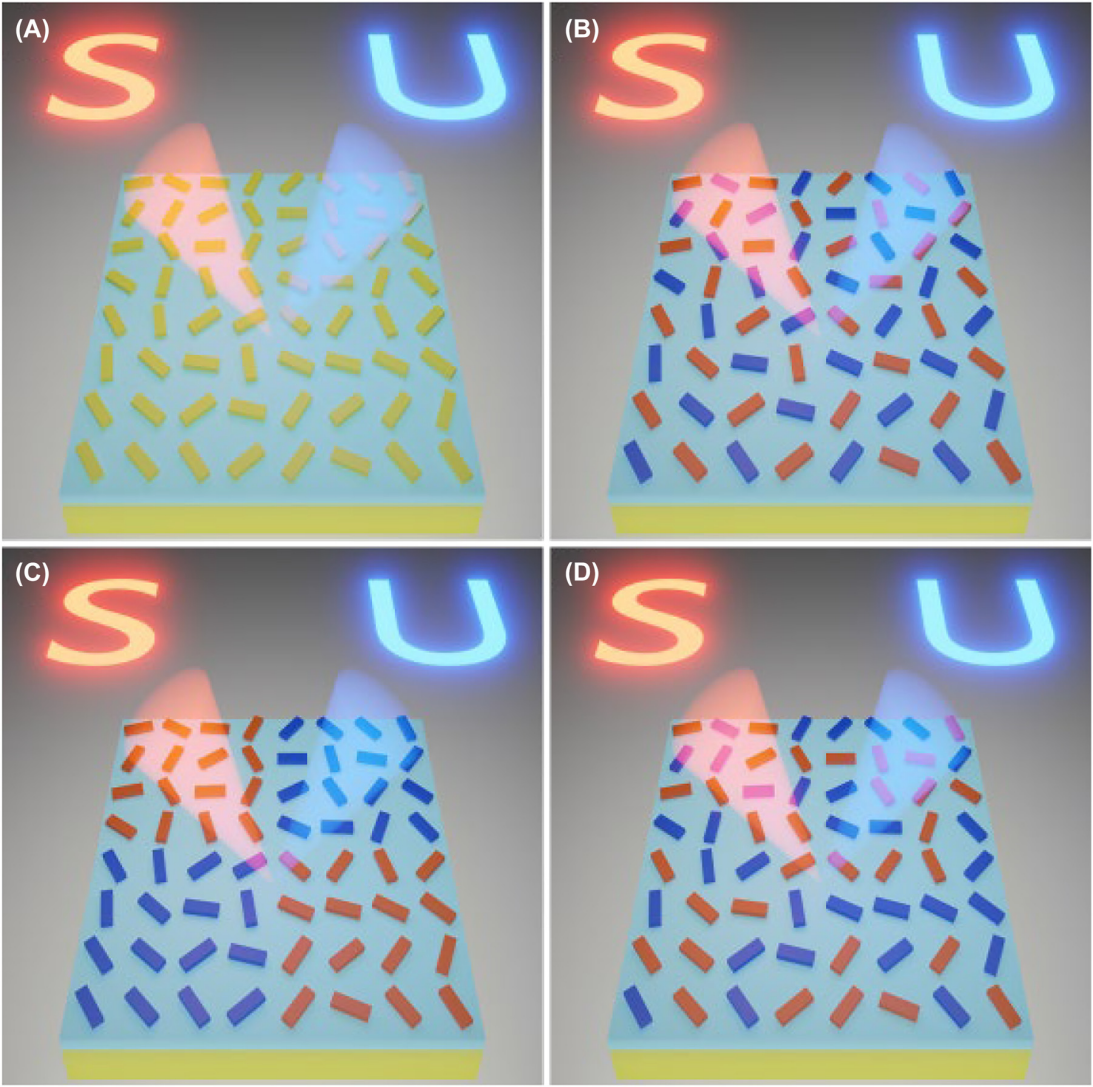
Schematic illustration of different design strategies for spin-multiplexed holograms by utilizing single-celled (A) non-interleaved, (B) interleaved, (C) segmented, and (D) randomly interleaved metasurfaces. The LCP and RCP incident beams get reflected from the geometric metasurface to form the holographic images “S” (red) and “U” (blue) in the far field.

Four different design strategies for spin-multiplexed meta-hologram based on single-sized meta-atoms that function as highly-efficient HWPs are considered for ensuring the geometric phase modulation in a broadband wavelength range as schematically illustrated in Figure 1. Under the illumination of LCP and RCP light beams, the holographic images “S” and “U” are reconstructed in the far field with orthogonal circular polarization states, respectively. To avoid overlap with the zero order, the holographic images are shifted away from the center and projected on the left and right sides with respect to the direction of the incident beam. The first design approach is to use a non-interleaved metasurface (NIMS), where the orientation angle of each meta-atom is determined by finding the optimal compromise between the two phases for the two holograms (Figure 1A). Other possibilities exploit the spatial degrees of freedom by using interleaved or segmented metasurfaces [25], [26], [27], [28], [29], [30]. As shown in Figure 1B, the interleaved metasurface (IMS) consists of a complicated unit cell with incorporated elements responsible for each holographic image, forming chessboard patterns of different sizes (i.e., 1 × 1 in Figure 1B). For the segmented metasurface (SMS) composed of four segments (Figure 1C), each quarter-hologram segment projects the light with a distinct spin in the far field. As an alternative, a randomly interleaved metasurface (RIMS) is proposed to avoid undesired diffraction spots by spatially arranging the meta-atoms responsible for two holograms in a random manner (Figure 1D). Compared to the NIMS with a compromised phase profile, the interleaved and segmented metasurfaces could supply ideally matched phases for both holographic images but at the expense of enlarged pixel sizes and reduced functional area for each spin channel along with the increased crosstalk-induced background.

**Figure 2:**
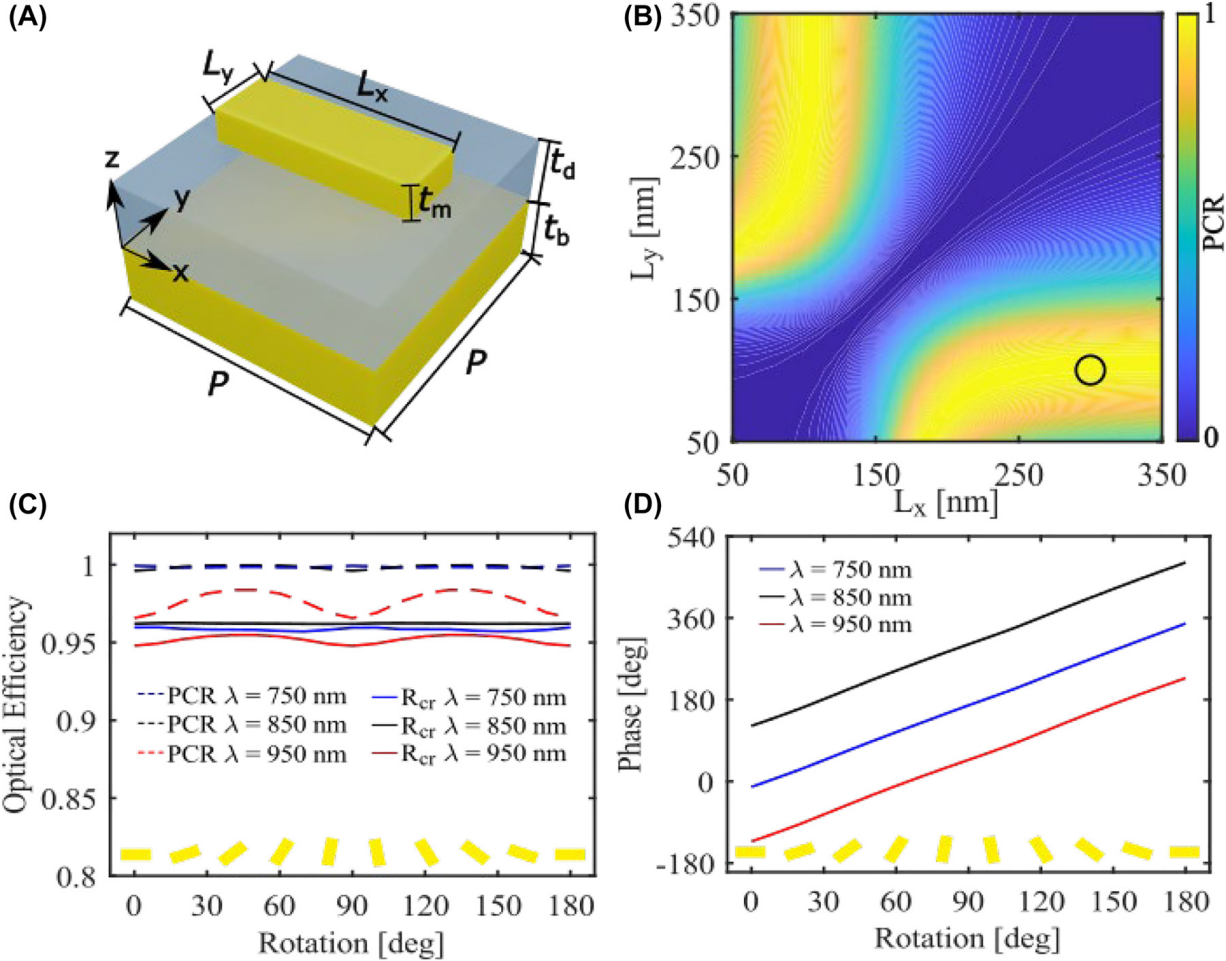
Simulation results of the GSP meta-atom. (A) The schematic of the GSP meta-atom. (B) The simulated PCR as a function of the lateral dimensions *L*_x_ and *L*_y_ at *λ* = 850 nm with the fixed periodicity of *p* = 400 nm, nanobrick thickness of *t*_m_ = 50 nm, SiO_2_ spacer thickness of *t*_d_ = 100 nm, and bottom Au film thickness of *t*_b_ = 100 nm. The black circle marks the dimension area with a high PCR above 96%. (C) The simulated PCR and *R*_cr_ of the desired meta-atom with different rotations at three different wavelengths. (D) The simulated phase shift of the cross-polarized reflected light of the desired meta-atom with different rotations at three different wavelengths under the RCP excitation. The orientation of the nanobrick is annotated at the bottom in (C) and (D).

After introducing different strategies for designing spin-multiplexed holograms, we simply utilize the anisotropic gap-surface plasmon (GSP) meta-atom as the fundamental building block to encode the geometric phase profile [8, 9, 31, 32], which ensures dispersionless phase modulation of the cross-polarized CP beams with high reflection efficiency, thereby alleviating the need for complex meta-atoms. As shown in Figure 2A, the GSP building block consists of a gold (Au) nanobrick on top of a silicon dioxide (SiO_2_) coated Au film. To ensure high efficiency and broadband operation, each anisotropic meta-atom is designed to act as a local HWP that fully converts a CP beam into the cross-polarized counterpart in reflection over a wide wavelength range. The meta-atom was simulated using the commercially available software COMSOL (ver. 5.6), where periodic boundary conditions were used in *x*- and *y*-directions and a perfectly matched layer was added to truncate the air domain on top of the meta-atom. A parameter sweep was done to find suitable lateral dimensions (i.e., *L*_x_ and *L*_y_) for the nanobrick while the other geometrical parameters were fixed. The meta-atom with lateral dimensions of *L*_x_ = 300 nm and *L*_y_ = 100 nm is selected for the unity polarization conversion ratio (PCR) at the design wavelength of *λ* = 850 nm (Figure 2B), which is defined as the ratio of the cross-polarized reflectivity (*R*_cr_) to the sum of cross- and co-polarized reflectivities (*R*_cr_ + *R*_co_) under the CP excitation. Meanwhile, the meta-atom exhibits a high efficiency with the cross-polarized reflectivity approaching 93% due to the suppressed optical loss. More simulation results (Figures S1 and S2) show that the designed meta-atom has a continuously high PCR above 93% for a bandwidth larger than 400 nm around the design wavelength of 850 nm with the corresponding cross-polarized reflectivity exceeding 85%, indicating the desired highly efficient broadband behavior. To verify the physical mechanism behind the wideband and efficient operation, the reflection amplitudes and phase shifts under linear-polarized excitations were simulated as a function of the wavelength ranging from 600 to 1100 nm (Figure S3). The meta-atom imparts a relative phase delay of ∼180° between the *x*- and *y*-polarized components in the investigated wavelength range of 700–1000 nm. At the same time, the reflection amplitudes are almost equal at high levels over 0.94. As such, the meta-atom functions as an efficient HWP to convert the incoming CP light into the cross-polarized counterpart in reflection. Moreover, the HWP functionality of the designed meta-atom is maintained despite the orientation of the topmost Au brick (Figure 2C). The tiny change in the optical efficiencies can be explained by the intrinsically weak near-field coupling of neighboring meta-atoms in our design. By rotating the meta-atom with an angle of *θ*, the phase shifts of 2*θ* are achieved for the cross-polarized CP light at all studied wavelengths (Figure 2D), verifying the broadband geometric phase modulation.

**Figure 3:**
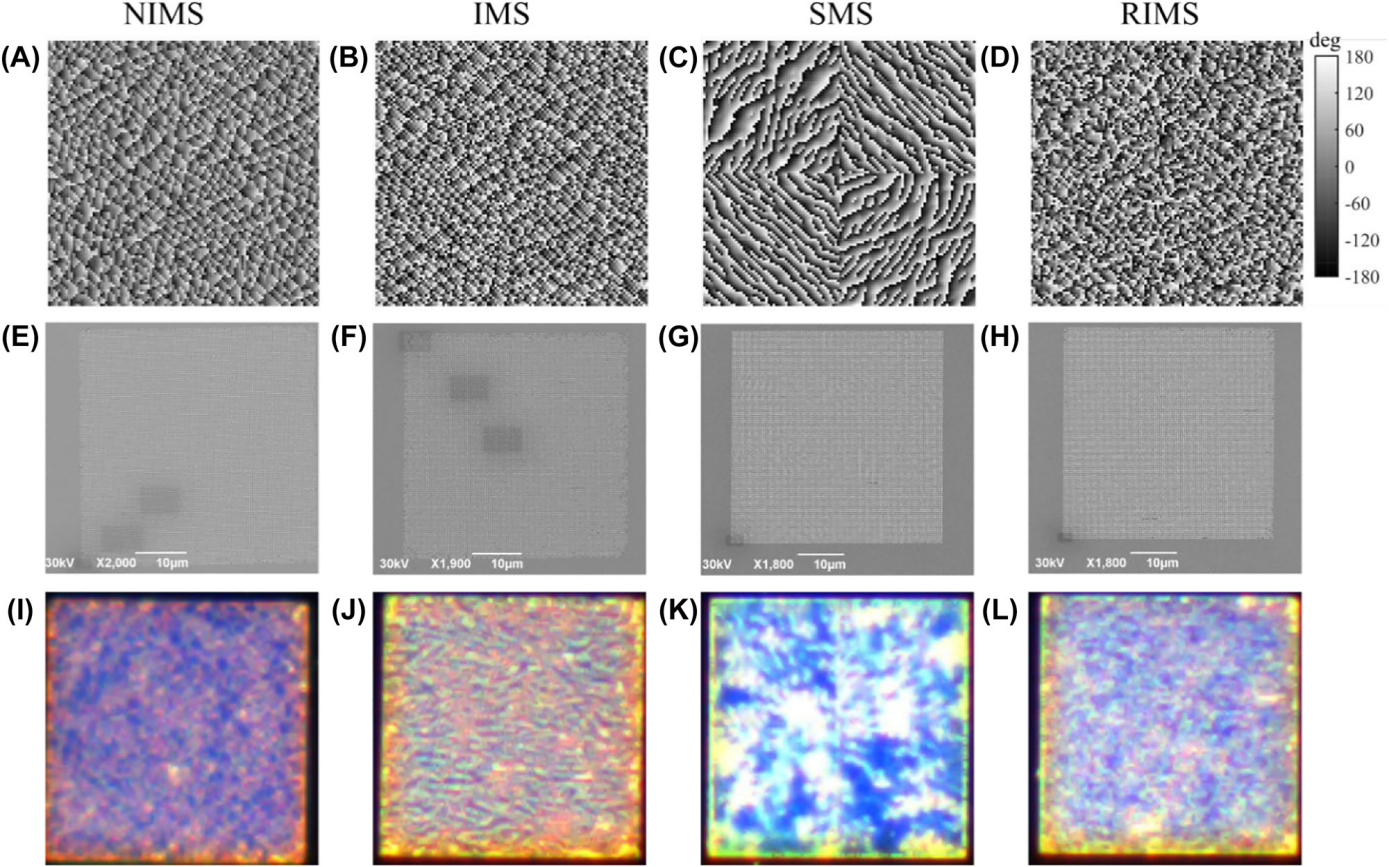
Four different metasurfaces for spin-multiplexed holograms. (A–D) phase profiles. (E–H) SEM images. (I–L) Darkfield microscope images.

The phase distribution required for the two holographic images was found with the standard Gerchberg–Saxton algorithm [33], which was implemented with 15 iterations for each design to ensure near-unity correlations between the target and reconstructed images (Figure S4). A minor amount of speckle noise is introduced by the phase retrieval of the Gerchberg–Saxton algorithm (Figure S5). Each metasurface consists of 111 × 111 pixels corresponding to an area of 44.4 × 44.4 µm. As shown in Figure 3C, the segmentation for the SMS is already visible from the phase map, which is different from the interleaved and non-interleaved designs. The designed four different metasurfaces were then fabricated using the thin-film deposition, electron beam lithography, and lift-off processes (see the Experimental Section for detailed information). Judging from the scanning electron beam (SEM) microscope images shown in Figuress 3E–H and Figure S6A, the four designs exhibit very similar distributions with spatially-oriented meta-atoms. However, the darkfield microscope images indicate distinct scanning features (Figure 3I–L). For instance, the four sectors in the SMS are observed (Figure 3K) while the other three designs show randomly distributed patterns. The NIMS darkfield image is smoother compared to IMS and RIMS, which is in line with the smoother phase distribution. This is expected as it is created by compromising the two possible phase choices for each meta-atom.

**Figure 4:**
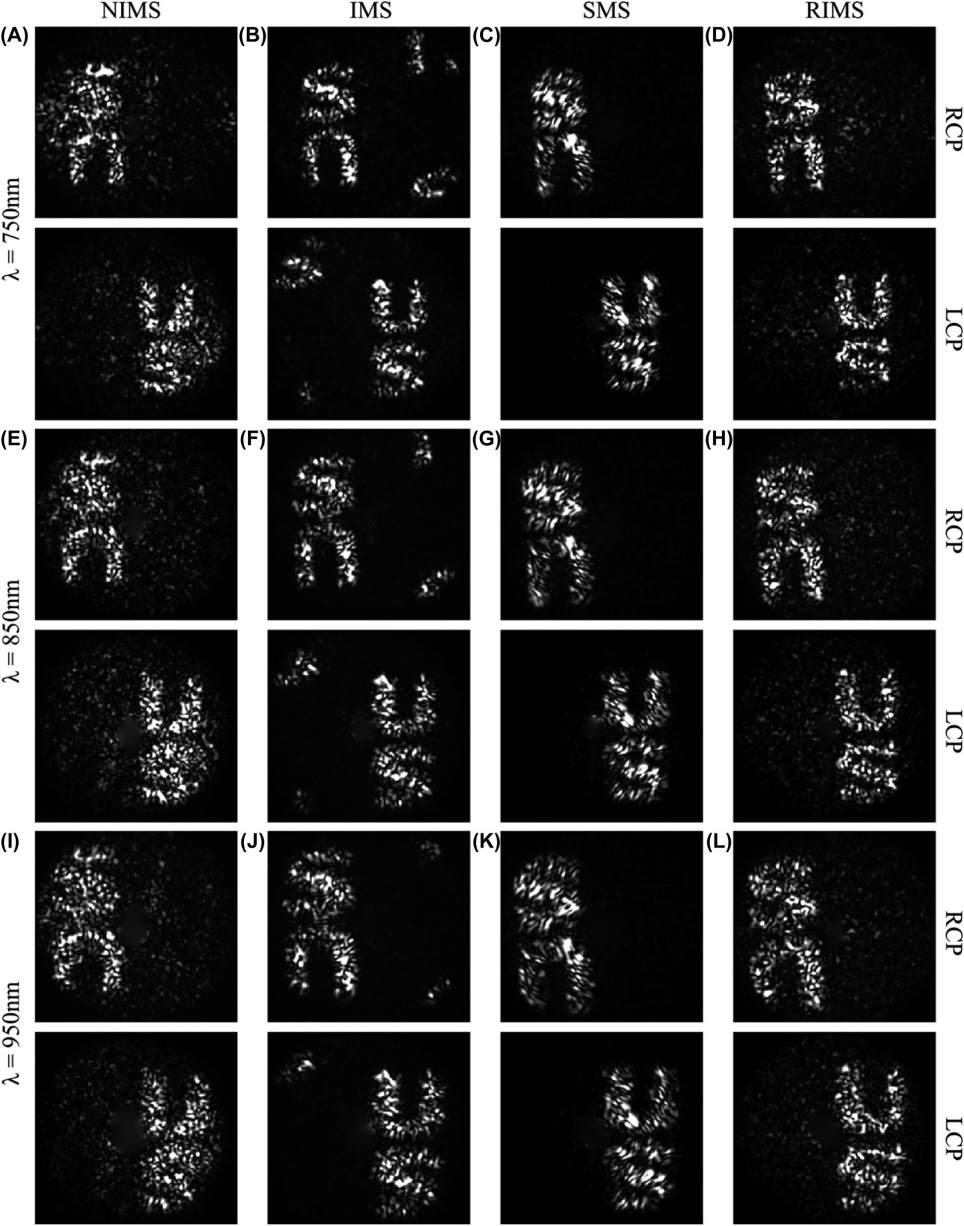
Reconstructed images of four different metasurfaces at wavelengths of (A–D) 750, (E–H) 850, and (I–L) 950 nm, respectively, under the RCP excitation (top) and LCP excitation (bottom).

After fabrication, we measured the holograms in the far field with a homemade microscope (see the Experimental Section and Figure S6B for detailed information). As shown in Figure 4, two holographic images can be well reconstructed for all four metasurfaces when illuminated by either an RCP or LCP light beam. For example, a letter “S” and a flipped letter “U” can be observed for the RCP excitation. Once the incident CP light is switched to the LCP beam, a flipped letter “S” and a letter “U” are reconstructed instead. This flipped twin image is ascribed to the conjugated nature of the geometric phase, which can change the sign of the phase profile on the metasurface plane when the incident spin is flipped. In this way, the intensity distribution of the target image *I*(*x*, *y*) becomes *I*(−*x*, −*y*) and these two images are centrosymmetric. To fully eliminate the twin image, additional degrees of freedom must be included in the phase modulation method, such as using a point source with varied locations [34] or utilizing the spatial freedom of the pixeled meta-atom [35, 36]. A change of incident spin can therefore be seen as a mirror operation and both holographic images are always visible. For each incident CP beam, the vivid reconstructed image can be clearly seen in a 290 nm bandwidth centered on the design wavelength of 850 nm without any significant changes, which is expected from the broadband operation of the designed HWP meta-atom. The main difference is size scaling in the reconstructed image, where a bigger hologram appears at a longer wavelength. This also leads to a slight blurring of the hologram under the illumination of a wideband light source (Figure S7).

Even though all four metasurfaces show reasonable performance, the reconstructed images are quite different in terms of background, contrast, and efficiency. The resulting holographic image of the NIMS shows a noisy background and blurry without any clear boundaries, which results from the nonideal encoded phase profile. Unlike the NIMS, the IMS could resolve a vivid hologram with suppressed backgrounds and clear boundaries. However, higher-order diffraction is visible at the edges of the field of view due to the interleaved pattern with an enlarged periodicity, especially at the short wavelength (Figure 4B). This higher-order diffraction would be out of the field of view at longer wavelengths, which is nearly the case in Figure 4J. To eliminate the unwanted diffraction, SMS and RIMS designs could be used. Compared to the IMS design, the RIMS shows a slightly increased background noise level, which may be ascribed to the random algorithm we used. But the noise level is still lower than the NIMS design. The SMS achieves good results in terms of the suppressed background and higher fidelity but is sensitive to the alignment of the incident beam since each segment only encodes one hologram, which could lead to one channel nearly disappearing in the worst case. Choosing a larger supercell periodicity to form the interleaved chessboard pattern could remove the higher order diffraction of the IMS with a smaller periodicity and be less sensitive to the exact alignment of the beam compared to the SMS design (Figure S8). As a final comment, it should be mentioned that all holographic images possess more speckles compared to the calculated images (Figure S5). They are known as artifacts in metaholography and can be suppressed or even removed to a high degree by introducing a certain amount of decoherence [37].

**Figure 5:**
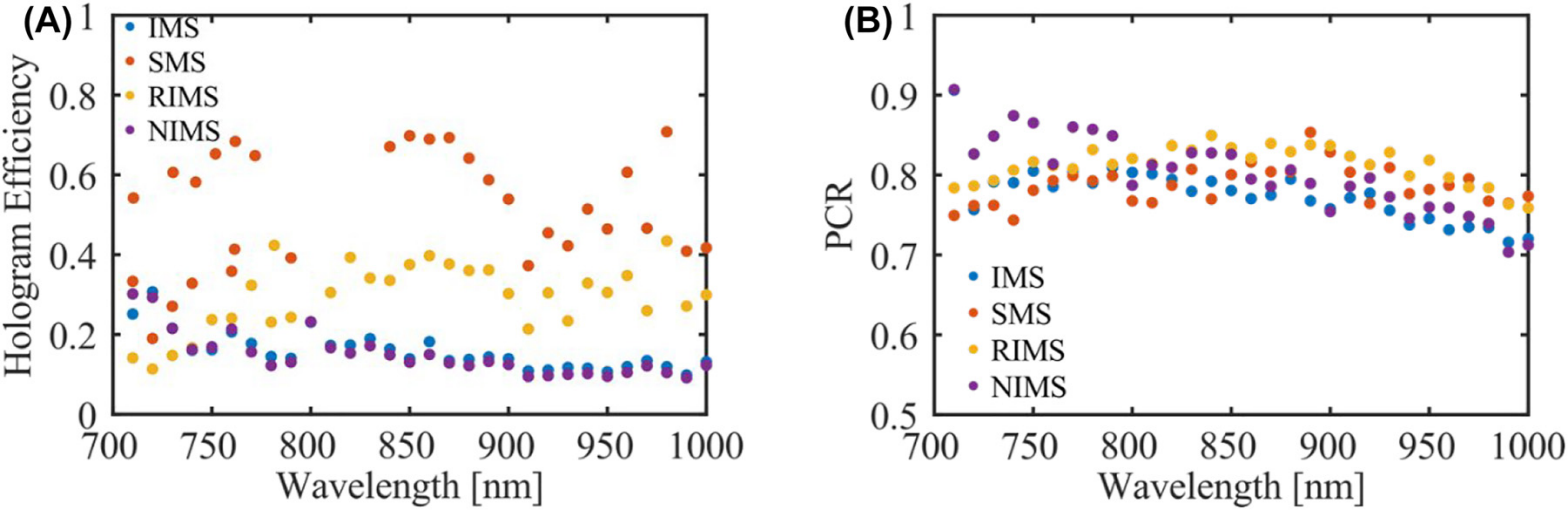
Measured (A) hologram efficiency and (B) PCR for the four different metasurfaces.

In order to quantitatively analyze the performance of all four metasurface holograms, the hologram efficiency, defined as the ratio of the power of the reflected light contained in the holographic images to the power of the incident light, was calculated by integrating the intensities in the captured images. As shown in Figure 5A, the SMS has the highest efficiency, consistent with the high quality of reconstructed images. As expected, the NIMS has a lower efficiency, which is mainly due to the higher background level originating from the nonideal phase profile. Owing to the unwanted high-order diffraction produced from the periodicity of the complicated unit cell, the IMS possesses similar low hologram efficiency, ascribed to the light directed into the higher order spots. To increase the efficiency of the IMS, a larger interleaving periodicity has been used for each pixel (Figure S5), which finally approaches the efficiency of the SMS if the periodicity is large enough. As a compromise, the RIMS achieves moderately high efficiency with acceptable noise levels (e.g., reduced background compared to NIMS and no high-order diffraction) and without strict alignment. Besides the hologram efficiency, the PCR of the holographic images has been measured and plotted in Figure 5B. Generally, all four metasurfaces exhibit similar and high polarization conversion ratios over the investigated spectrum range, consistent with the high PCR of the designed GSP meta-atom. The tiny difference may originate from fabrication impurities and slight alignment shifts during the measurements.

## 3 Conclusions

In this work, we have implemented four different single-celled metasurfaces for spin-dependent metaholography in a broadband spectrum ranging from 710 to 1000 nm. By studying the background levels, image fidelities, and efficiencies of reconstructed holographic images, we provided a systematic comparison between the non-interleaved, interleaved, and segmented meta-holograms, among which the SMS exhibits the superior performance of reduced background noise and higher hologram efficiency but at the expense of alignment constraint, which could be released by employing IMS with a larger interleaved periodicity, finally transiting to an SMS with more segments. The holographic image quality can be improved by fabricating larger metasurfaces that provide finer features in the far field. Additionally, advanced multiplexing method beyond spins, such as orbital angular momentum multiplexing [38, 39], can be introduced to increase the information capacity of multiplexed metaholograms. The demonstrated work could be a versatile methodology for designing general meta-holograms and be extended to encode mass information beyond spin-encoded channels by exploiting advanced c methods [40, 41].

## 4 Experimental section

### 4.1 Fabrication

The fabrication was conducted by combining the thin-film deposition, electron beam lithography, and lift-off processes. First, a 3 m Ti layer, a 100 nm Au layer, and a 3 nm Ti layer were successively deposited on a silicon substrate using thermal vapor deposition. Then RF sputtering was used to deposit a SiO_2_ spacer layer with a thickness of 100 nm. After that, an approximately 120 nm thick PMMA (2% in anisole, Micro Chem) layer was spin-coated on top of the SiO2 layer as an electron beam resist, which was then baked at 180 °C for 2 min and exposed at an acceleration voltage of 30 keV to define the meta-atoms. The exposed area was developed in the solution of methyl isobutyl ketone (MIBK) and isopropyl alcohol (IPA) of MIBK: IPA = 1:3 for 35 s and then fixing in a pure IPA stopper. Afterward, a 3 nm Ti layer and a 50 nm gold layer were deposited using thermal vapor deposition again, which was finally lifted off in acetone. The fabricated sample was evaluated using a scanning electron microscope.

### 4.2 Measurements

The measurements were conducted with a homebuilt optical setup (Figure S4B). The light from a Ti: Sapph laser (Spectra-Physics, Model 3900S) passes through first an attenuator and then a combination of a linear polarizer and a quarter wave plate to create the CP light with controlled intensity. The CP light passes through two identical beam splitters before reaching the sample through an objective (Nikon E Plan 100× /0.90 EPI). The tow beam splitters are used to compensate for the actual phase retardance caused by one single beam splitter. Furthermore, a lens (Lens 1, Thorlabs LA1608-B-ML) in between the beam splitters, in combination with the objective, produces plane wave incident on the sample. The reflected signal is collected by the same objective and then passes through a tube lens (Lens2, Thorlabs TTL200-S8) and an iris to select the region of interest in the fabricated sample. To filter out the different CP components, a linear polarizer and a quarter-wave plate have been added. Two more lenses (Lens3, Thorlabs AC254-125-B-ML and Fliplens4, Thorlabs LBF254-100-B) are used to switch between the direct and Fourier images captured by a CMOS camera (Thorlabs DCC1545M-GL). The direct image combined with a white light source is used to image the samples and position the laser spot, whereas Fourier images serve as the reconstructed Fourier images and image the holograms.

## Supplementary Material

Supplementary Material Details
