# Supplementary material for: Broadband spin-multiplexed single-celled metasurface holograms: a comprehensive comparison between different strategies
Source: Nanophotonics. 2023 Jan 9;12(8):1363–71. doi: 10.1515/nanoph-2022-0535 (PMC11501439; doi:10.1515/nanoph-2022-0535)
Supplement: Supplementary file 1 — Supplementary Material Details [file j_nanoph-2022-0535_suppl_001.pdf]

Supporting Information:

Broadband Spin-Multiplexed Single-Celled Metasurface Holograms: A Comprehensive Comparison between Different Strategies

Sören im Sande, Sergey I. Bozhevolnyi, and Fei Ding*

Centre for Nano Optics, University of Southern Denmark, Campusvej 55, Odense M DK-5230, Denmark

*Corresponding author: feid@mci.sdu.dk

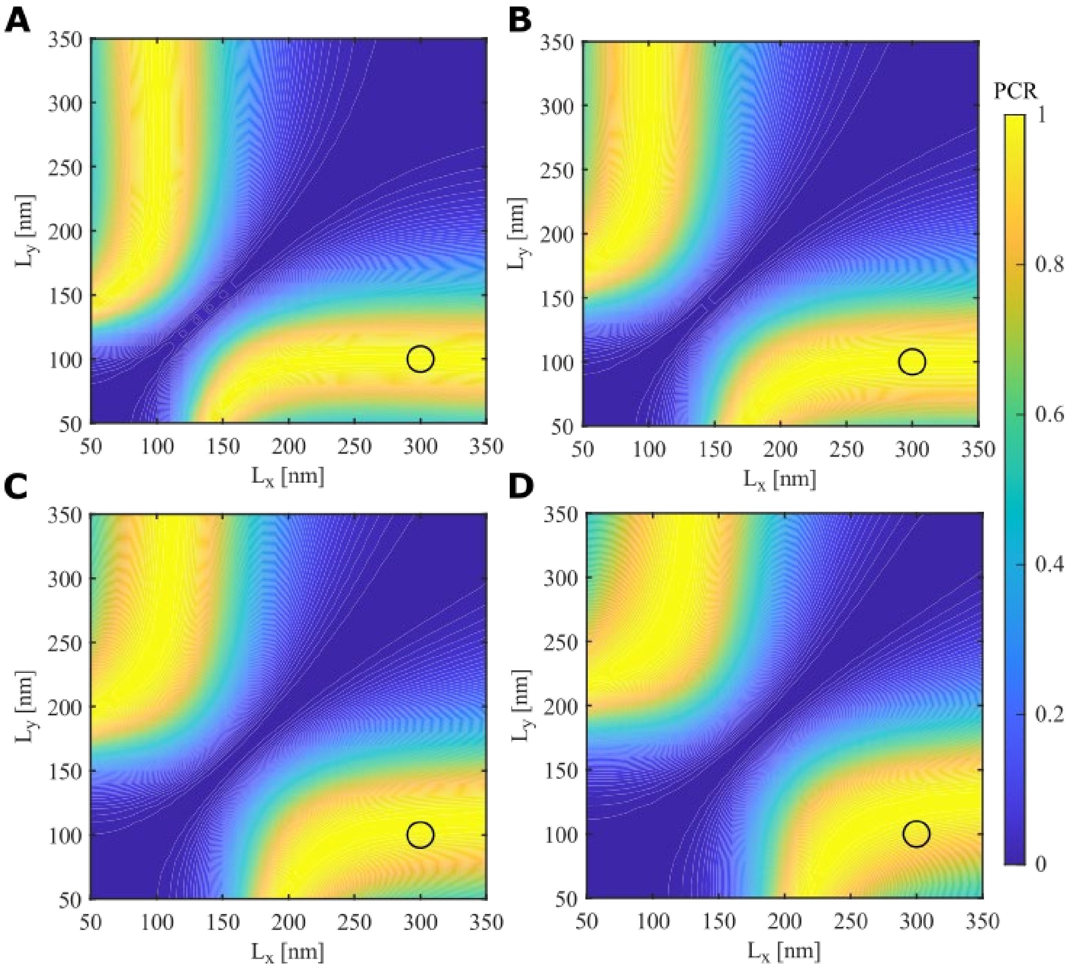

Figure S1: The simulated polarization conversion ratio (PCR) as a function of the lateral dimensions L_x and L_y with the fixed periodicity of P = 400 nm, nanobrick thickness of t_m = 50 nm, SiO_2 spacer thickness of t_d = 100 nm, and bottom Au film thickness of t_b = 100 nm at the wavelengths of (A) 700, (B) 800, (C) 900, and (D) 1000 nm. The black circle indicates a 10 nm difference around the selected dimensions of L_x = 300 nm and L_y = 100 nm.

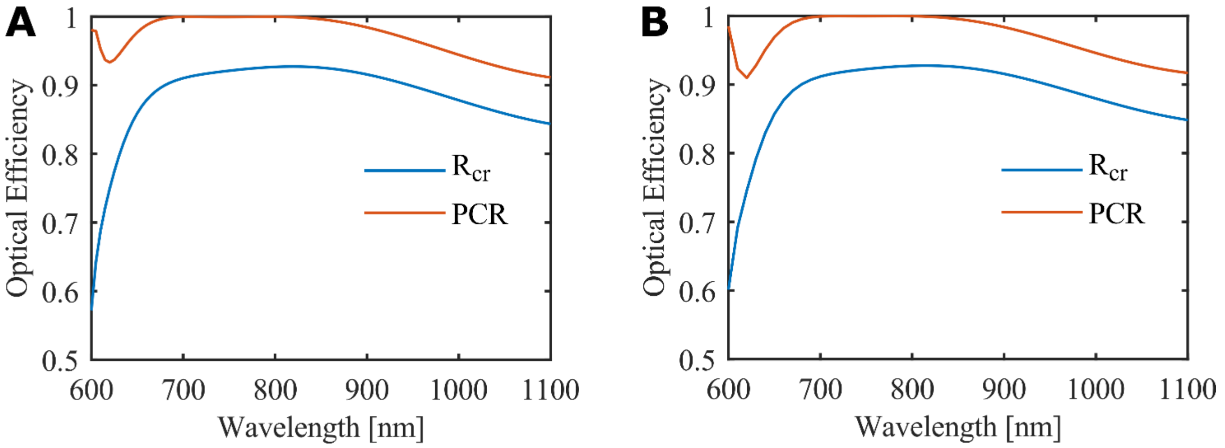

Figure S2: (A) Simulated cross polarized reflectivity and PCR as a function of the wavelength for the designed meta-atom. (B) Simulated cross polarized reflectivity and PCR as a function of the wavelength for the designed meta-atom whose corners are rounded with a 5 nm radius.

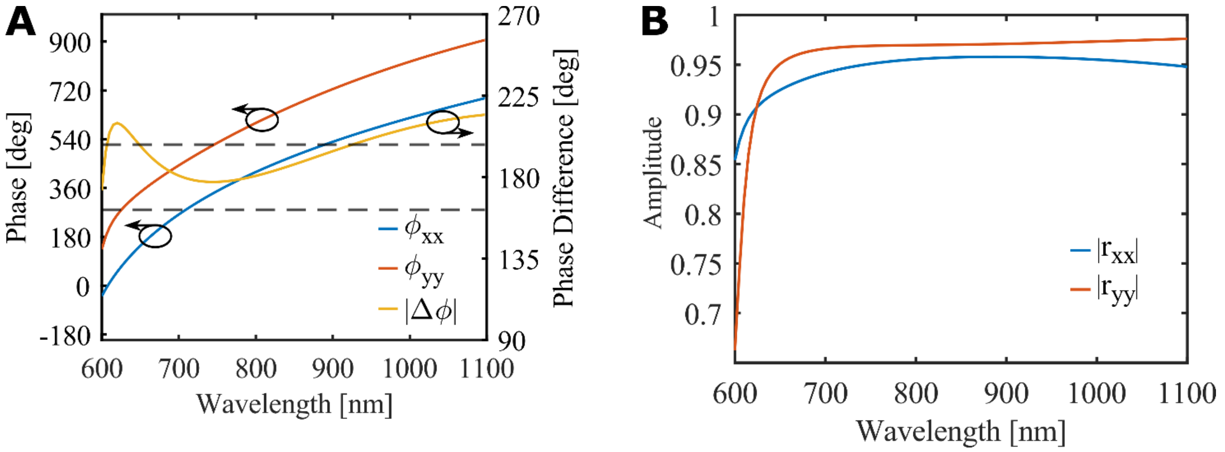

Figure S3: (A) Simulated reflection phases and relative phase difference as a function of the wavelength for *x*- and *y*-polarized light. The ±10% deviations from desired 180° phase difference are indicated by dashed black lines. (B) Simulated reflection amplitudes as a function of the wavelength for *x*- and *y*-polarized light.

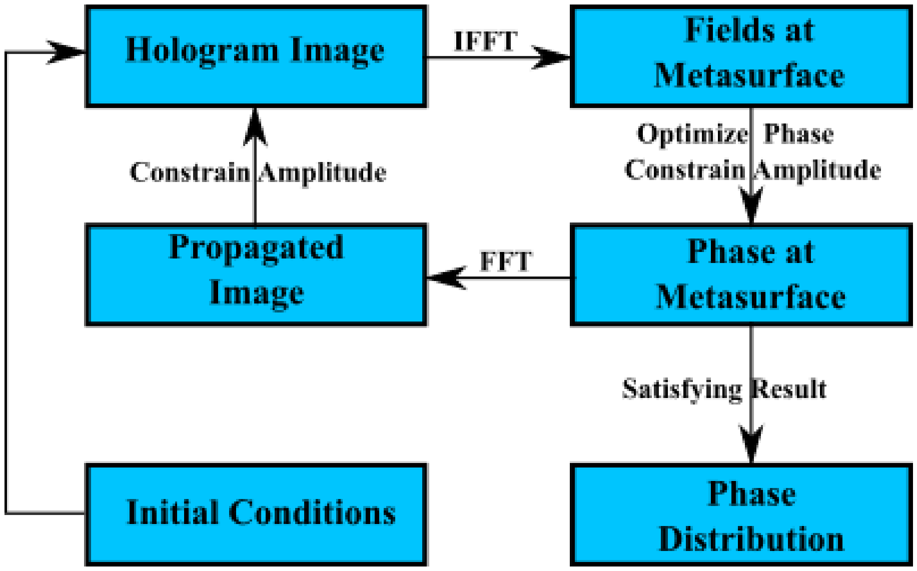

Figure S4: Flow diagram of the standard Gerchberg-Saxton algorithm. The algorithm starts with the amplitude distribution of the desired holographic image and a randomly assigned phase distribution. This holographic image is projected to get the fields at the metasurface using the inverse fast Fourier transform (IFFT) method. To design the compromised phase profile for the non-interleaved metasurface, the phase optimization is conducted by minimizing $|\theta(x_i, y_j) - \varphi_1(x_i, y_j)/2|^2 + |\theta(x_i, y_j) + \varphi_2(x_i, y_j)/2|^2$, where $\theta(x_i, y_j)$ represents the rotation angle of the meta-atom centered at the point (x_i, y_j), $\varphi_1(x_i, y_j)$ and $\varphi_2(x_i, y_j)$ stand for the required phases at the metasurface to reconstruct two images, and (x_i, y_j) is the local surface coordinate. The amplitude is constrained to uniform illumination and only the phase distribution along the metasurface is considered. The image is then back-propagated using a fast Fourier transform (FFT), whose amplitude is compared with the target image. If they match each other to a satisfying degree, the phase distribution is found and returned in the next cycle. Otherwise, the cycle is repeated.

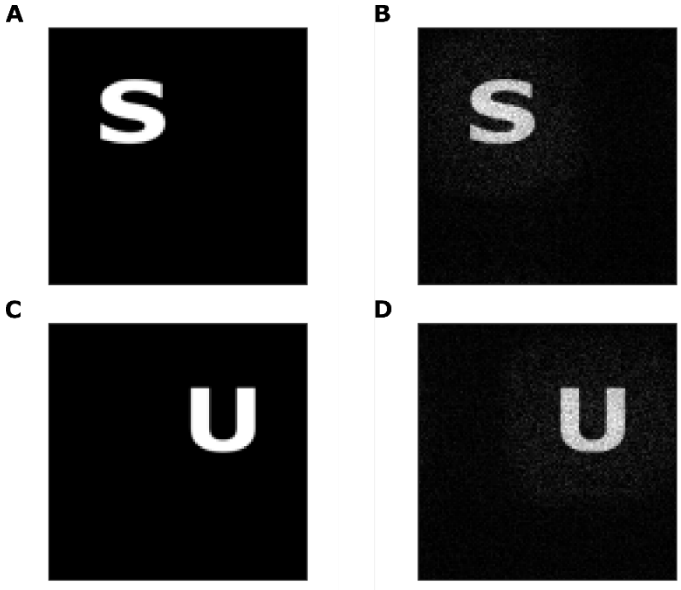

Figure S5: (A,C) Targeted and (B,D) reconstructed images with the Gerchberg-Saxton algorithm.

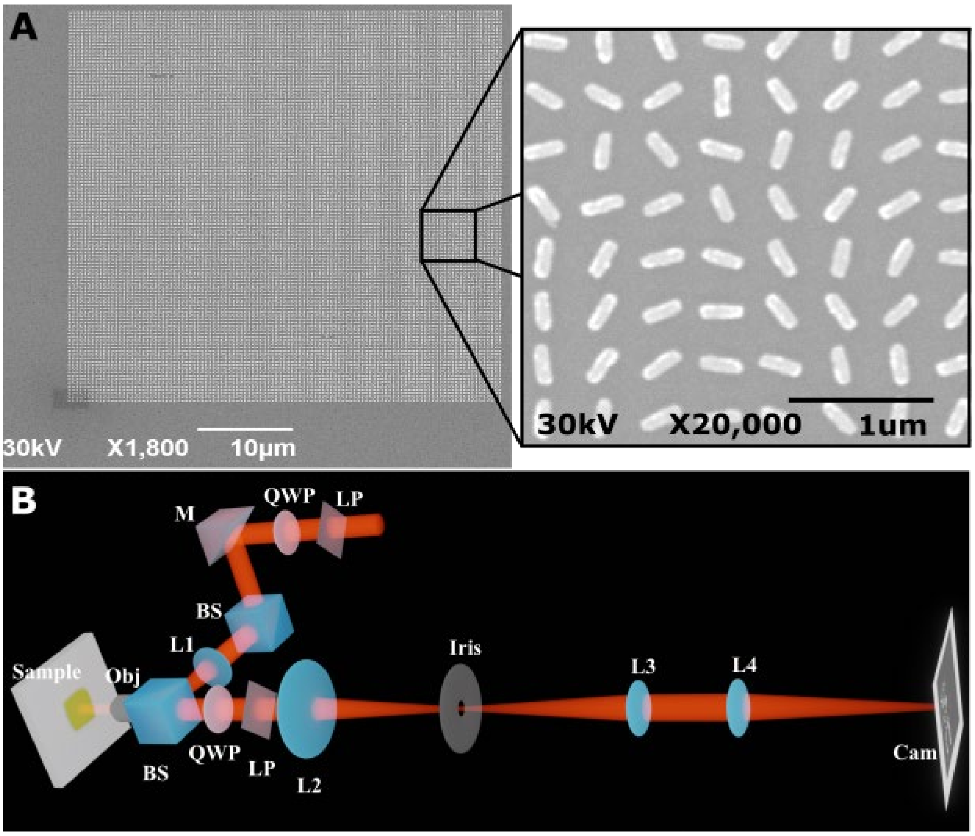

Figure S6: (A) SEM images of one fabricated metasurface. The rotation of the brick meta-atoms is clearly visible. (B) Schematic of the optical setup for the hologram measurement. LP: linear polarizer; QWP: quarter wave plate; M: mirror; BS: beam splitter; L1-L4: lenses; Obj: objective.

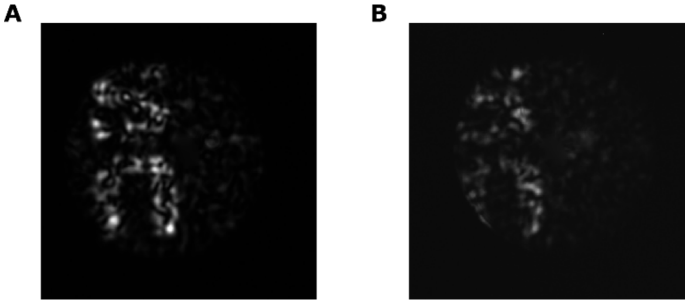

Figure S7: Reconstructed images of the segmented metasurface illuminated with a broadband light source with a (A) 10 nm and (B) 40 nm bandpass filters centered around the wavelength of 850nm.

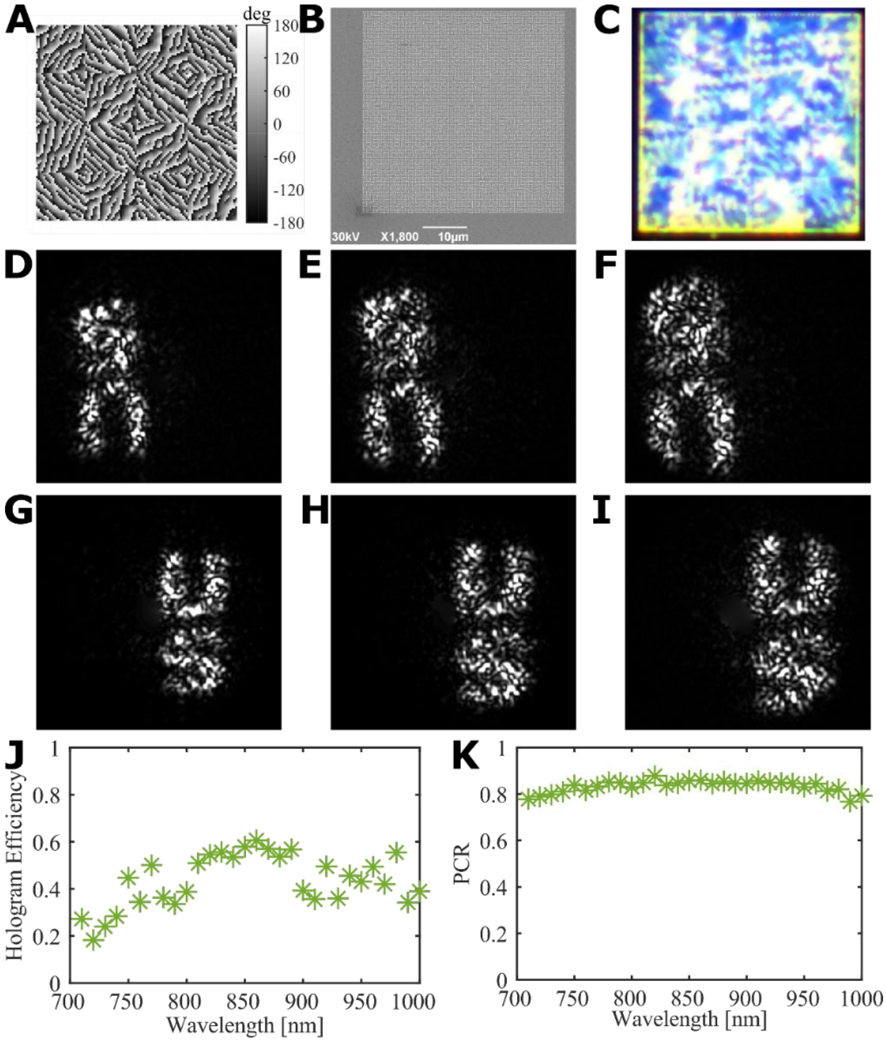

Figure S8: The design and performance of the interleaved metasurface with a larger periodicity of 10 μm (refereed as LIMS in the following). (A) Calculated phase distribution. (B) SEM image of the LIMS. (C) Darkfield microscope image. (D-I) Reconstructed images at wavelengths of (D,G) 750, (E,H) 850, and (F,I) 950 nm under the (D-F) RCP and (G-I) LCP excitations. Measured (J) hologram efficiency and (K) PCR for the four different metasurfaces.
